# ChatGPT: performance of artificial intelligence in the dermatology specialty certificate examination^☆^

**DOI:** 10.1016/j.abd.2023.08.005

**Published:** 2023-11-18

**Authors:** Thaís Barros Felippe Jabour, José Paulo Ribeiro, Alexandre Chaves Fernandes, Cecília Mirelle Almeida Honorato, Maria do Carmo Araújo Palmeira Queiroz

**Affiliations:** a Centro de Pesquisas Clínicas de Natal, Natal, RN, Brazil; b Department of Dermatology, Hospital Universitário Onofre Lopes, Universidade Federal do Rio Grande do Norte, Natal, RN, Brazil; c Instituto Internacional de Neurociências Edmond e Lily Safra, Macaíba, RN, Brazil; d Hospital Universitário Onofre Lopes, Universidade Federal do Rio Grande do Norte, Natal, RN, Brazil

*Dear Editor,*

ChatGPT (Chat Generative Pre-Trained Transformer), launched by OpenAI in 2022, is an advanced Artificial Intelligence (AI) language model capable of interacting in conversations and providing original responses. Despite great speculation, its use to aid clinical decision-making is still not recommended due to the lack of information about its technical knowledge in medicine and the potential ethical impacts involved.^1^, ^2^

As a first step towards obtaining this information, ChatGPT has been recently tested in specialty certificate examination tests for different medical specialties.^3^, ^4^, ^5^ In Brazil, few studies have been carried out in this regard, and to date none have evaluated its performance in the specialty certificate examination test in Dermatology (TED, *Título de Especialista em Dermatologia*, in Portuguese).

TED is obtained by taking an annual examination offered by the Brazilian Society of Dermatology (SBD, *Sociedade Brasileira de Dermatologia*), with the first eliminatory phase consisting of a theoretical test comprising 80 objective questions, with four alternatives each and only one correct option. Candidates who get less than 60% of correct answers (48 correct questions) are eliminated from the assessment.^6^ The authors carried out this study aiming to evaluate the performance of this AI in this examination and considering its use in dermatology.

The performance of ChatGPT (version 4) was evaluated by solving the TED 2022 and 2023 questions, available on the SBD website. Questions with images were disregarded, due to the absence of this functionality, and were canceled. The questions were classified into clinical, laboratory, surgical and cosmetic dermatology. The application was instructed to answer multiple-choice questions. Then, one question was presented at a time so that it could choose the most appropriate alternative. Overall accuracy was measured as the ratio between the number of correct answers and the total number of questions. Accuracy was also evaluated per year of the test and for each area, comparing the average of the tests using the Student's *t* test or Mann-Whitney test, depending on the normality of the data, which was verified using the Shapiro-Wilk test. Subsequently, the variation in performance throughout the test was assessed by grouping all the questions into sets of ten, following the test order. In each group, the percentage of correct answers was calculated, disregarding canceled questions and those with images in that group. Spearman's test was used to evaluate the correlation between the set percentage of correct answers and the number of the initial questions in the respective set, as well as a graphic tendency line using linear regression, seeking to identify whether there was an improvement in performance throughout the test.

A total of 146 questions were analyzed, 74 questions from TED 2023 and 72 from TED 2022. Nine canceled questions were excluded, as well as five that contained images. The overall accuracy was 75.34%, with the average performance in the 2022 test being higher than that in 2023 (Fig. 1). In the statistical evaluation for comparison between the examinations, as there was no normality of the data, the Mann-Whitney test was performed, with a p-value of 0.29. The area with most correct answers was cosmetics and the one with the fewest was surgery (Table 1). The tendency lines show a positive slope, suggesting improved performance throughout the test in both years (Fig. 2). Spearman's correlation analysis showed a positive and moderate, albeit non-significant, correlation for TED 2022 (correlation = 0.43, p = 0.28) and TED 2023 (correlation = 0.32, p = 0.43).

**Figure 1:**
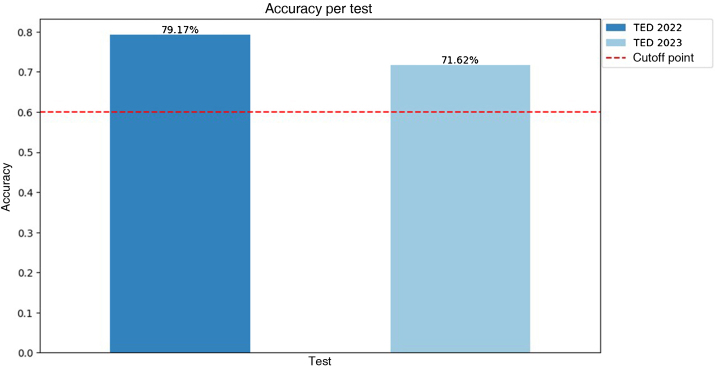
ChatGPT accuracy per test compared to the cutoff point for passing the examination.

**Table 1:** Percentage distribution of correct answers per area and year, with the total number of questions in parentheses.

Area	Total	2022	2023
Surgery	50 (4)	0 (1)	66.67 (3)
Clinics	77.87 (122)	86.67 (60)	69.35 (62)
Cosmiatry	80 (5)	50 (2)	100 (3)
Laboratory	60 (15)	44.44 (9)	83.33 (6)
**Total**	75.34 (146)	79.17 (72)	71.62 (74)

**Figure 2:**
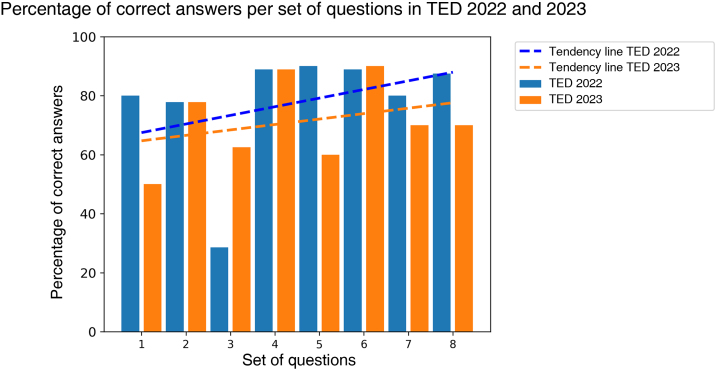
Percentage of correct answers per sets of questions throughout the test in TED 2022 and 2023, suggesting improved performance as the tool was used.

The ChatGPT performance in the 2022 and 2023 TED examinations was above what is required to obtain approval in the first phase of the competition. However, a comparative assessment of annual performances showed no statistically relevant difference, preventing determination of superiority of one year over the other. The performance analysis throughout the test suggests a tendency for the probability of correct answers to increase as more questions are answered, although more questions are necessary to reach a more significant statistical value. The categories with the best performance were cosmetic and clinical dermatology, suggesting a predominance of these areas in the ChatGPT training database.

The results were in line with other experiments carried previously in other medical examinations. A recently published article demonstrated that ChatGPT performs on par with a third-year medical student when answering the United States Medical Licensing Examination (USMLE) questions.^3^ In dermatology, the application has already been used to solve questions on the specialty certification test in the United Kingdom. In the study, ChatGPT-3.5 obtained an overall score of 63.1% and ChatGPT-4 obtained 90.5%. The expected passing score for the Dermatology SCE is 70%‒72%.^4^

Although the results are promising, there are yet no studies to support the use of ChatGPT in medical practice.^2^ A major limitation of its application in dermatology is its failure to evaluate images, considering the visual nature of the specialty. Caution is needed when incorporating AI into dermatological practice, and it is essential to carry out new studies in collaboration with experts in dermatology and AI to improve the understanding of its potential risks and benefits. A thorough clinical examination and a good doctor-patient relationship remain, to date, the best tools for a safe, reliable and effective clinical practice.

## Financial support

None declared.

## Authors' contributions

Thaís Barros Felippe Jabour: Design and planning of the study; collection, analysis and interpretation of data; critical review of the literature; drafting the manuscript or critical review of important intellectual content.

José Paulo Ribeiro Júnior: Collection, analysis and interpretation of data; critical review of the literature; drafting the manuscript or critical review of important intellectual content.

Alexandre Chaves Fernandes: Design and planning of the study; statistical analysis; drafting the manuscript or critical review of important intellectual content.

Cecília Mirelle Almeida Honorato: Critical review of the literature; drafting the manuscript or critical review of important intellectual content.

Maria do Carmo Araújo Palmeira Queiroz: Critical review of the manuscript; approval of the final version of the manuscript.

## Conflicts of interest

None declared.
